# Plasmonic nanodisc arrays on calcinated titania for multimodal analysis of phosphorylated peptides†

**DOI:** 10.1039/C7RA08870A

**Published:** 2017-10-12

**Authors:** Samuel S. Hinman, Romie C. T. Nguyen, Quan Cheng

**Affiliations:** a Environmental Toxicology, University of California - Riverside, Riverside, CA 92521, USA quan.cheng@ucr.edu; Fax: +1-951-827-4713; Tel: +1-951-827-2702; b Department of Chemistry, University of California - Riverside, Riverside, CA 92521, USA

## Abstract

A hybrid material of gold nanodiscs on a calcinated titania nanofilm that allows for selective quantitative and qualitative characterization of surface-enriched phosphopeptides has been designed and reported. Fabrication was realized through a combination of layer-by-layer deposition and high temperature calcination for the titania, and hole-mask colloidal lithography for the plasmonic nanostructures. The morphology of the resulting titania material was rigorously characterized, exhibiting substantially decreased surface roughness, which allows for lithographic fabrication of plasmonic nanostructures. Moreover, high specificity in adsorption and enrichment of phosphopeptides was exhibited, which was verified by LSPR shifts and matching peaks under mass spectrometric analysis. The construction of these biochips should inform other combinatorial nanofabrication techniques, in addition to allowing future phosphoproteomic analyses to be performed in a time and resource-efficient manner.

## Introduction

The post-translational modification (PTM) of proteins represents a set of processes that greatly increase the diversity of biological structures and functionalities within the cellular proteome. Phosphorylation of proteins is one PTM that carries broad impacts throughout cellular signaling, growth, homeostasis, and disease,^1^ though the reversible and dynamic nature of this modification render it difficult to isolate and study. Mass spectrometry has become the ideal tool for the study of this PTM, as it exhibits high sensitivity, throughput, and the ability to specifically identify phosphorylated amino acid residues.^2^ However, due to the low abundance of phosphopeptides within biological samples, enrichment processes have become routine within sample processing.^3^ Metal oxide affinity chromatography (MOAC) has emerged as a powerful method for phosphopeptide enrichment over the past several years, and relies on high affinity bidentate interactions between phosphate groups and the metal ions displayed at metal oxide surfaces.^4,5^ While titania (*i.e.* TiO_2_ or titanium dioxide) is the most widely used MOAC agent, having been incorporated into column,^6,7^ plate,^8–10^ pipette tip,^11^ and membrane^12^ formats, the use of zirconia, alumina, and tin dioxide has also been demonstrated.^5,13–15^ Fabrication techniques for these platforms have varied, from benchtop methods to cleanroom processes, with the synthetic conditions suggested to play an important role in the characteristics and performance of the final material.^5^

While mass spectrometric analyses of phosphorylated species have been highly successful with MOAC, increasing the informational power through complementary, real-time sensing techniques is desirable. Localized surface plasmon resonance (LSPR) spectroscopy is one method poised for ideal integration with on-plate MOAC and matrix-assisted laser desorption ionization/mass spectrometry (MALDI-MS). LSPR refers to the collective oscillation of delocalized electrons around a nanoparticle (*e.g.* gold or silver) that is much smaller than the wavelength of the incident light source responsible for excitation.^16^ The manifestation of localized surface plasmons is typically monitored through the presence of an absorbance band within the extinction spectrum of the nanomaterial, which the majority of laboratories are equipped to measure. The location and shape of this absorbance band are heavily dependent on the shape, periodicity, and composition of the nanomaterial, in addition to the bulk refractive index of the surrounding media.^17^ This latter dependency grants the ability to conduct label-free and real-time sensing within close vicinity of LSPR active nanostructures, both in solution and surface bound formats, given that the surface chemistry of the nanostructures is tailored for target-specific capture.^18^ The integration of LSPR and mass spectrometry has previously been demonstrated for matrix-assisted ionization of amyloid-beta derived diffusible ligands,^19^ and surface-assisted ionization of peptides and small molecules.^20,21^ In each of these studies, LSPR provided quantitative real-time binding information, while commercial MALDI mass spectrometers were used to confirm the identity of target compounds on-chip. Similar studies focused on LSPR detection have been attempted for titania-based MOAC, using gold nanoparticles deposited onto commercial glass slides, which were thereafter spin-coated with a titania (*i.e.* titanium isopropoxide) solution.^22,23^ While effective for sensitive phosphoprotein detection, interests have gradually shifted toward lithographically fabricated nanostructures due to their reproducibility and ability to manufacture in quantity,^24^ thereby paving the path for more accessible high-throughput analyses to be conducted.^25,26^

We have previously demonstrated that the layer-by-layer technique combined with a high temperature calcination process (LbL/calcination) is suitable for the benchtop fabrication of nanoscale titania films with controllable thickness.^9^ Rough surface features, such as microscale pores and aggregates, contributed to the overall surface area and were beneficial for phosphopeptide loading and MALDI-MS analysis, though these features prevent the uniform lithographic fabrication of superimposed nanostructures. The method of application (*e.g.* immersion, spraying, *etc.*) for LbL/calcination has been shown to have profound impacts on the final surface morphology for silicate nanofilms,^27,28^ which we investigate herein for calcinated titania. Utilizing an immersion based application of each titania precursor layer resulted in a substantially reduced surface roughness over previously utilized spray techniques,^9^ rendering the surface amenable to direct, lithographic nanoparticle fabrication techniques. We thereafter applied arrays of gold nanodiscs (AuNDs) through hole-mask colloidal lithography (HCL) to the calcinated titania films, which exhibited excellent uniformity in morphology and optical characteristics. The titania/AuND substrates were capable of on-chip enrichment of phosphopeptides from a tryptic digest of β-casein, as confirmed by MALDI-MS and LSPR. We anticipate the methods developed here to inform future combinatorial nanofabrication techniques, in addition to the material itself becoming part of the growing toolkit for phosphoproteome analysis.

## Experimental section

### Materials and reagents

β-Casein, trypsin (from bovine pancreas), anisole, poly(methyl methacrylate) (PMMA, MW ~ 996 kDa), poly(allylamine hydrochloride) (PAH, MW ~ 56 kDa), titanium(IV)bis(ammo-niumlactato)dihydroxide solution (TALH, 50 wt% in H_2_O), trifluoroacetic acid (TFA, 99%), Super-DHB, and *n*-octadecyltri-chlorosilane (C18, 90%) were from Sigma-Aldrich (St. Louis, MO). Ethanol (200 proof) and acetonitrile (ACN) were from Fisher Scientific (Pittsburgh, OH). Phosphoric acid (85% w/w) was from EMD Millipore (Billerica, MA). Poly(-diallyldimethylammonium chloride) solution (PDDA, 20%) and carboxylated polystyrene nanospheres (0.2 μm, 2.6% solids) were from Polysciences, Inc. (Warrington, PA). BK7 glass substrates were from Corning (Painted Post, NY). Chromium and gold used for electron-beam evaporation were acquired as pellets of 99.99% purity from Kurt J. Lesker (Jefferson Hills, PA). Nanopure water (≥18 MΩ cm), purified through a Barnstead E Pure filtration system (Thermo Scientific, Rockford, IL), was used for all reagent preparations.

### Instrumentation

Absorbance spectra were collected using a USB 2000 + UV-Vis spectrometer with illumination from a HL-2000 Tungsten-Halogen light source guided through 200 μm optical fibers (Ocean Optics, Dunedin, FL). Mass spectra were acquired as an average of 60 laser shots using a Voyager-DE STR MALDI-TOF mass spectrometer (Applied Biosystems, Framingham, MA) operating in positive reflector mode at an accelerating voltage of 20 kV. Scanning electron microscopy (SEM) was performed on an FEI NNS450 SEM (Hillsboro, OR) in CFAMM at UC Riverside. For SEM analysis, all samples were sputtered with a Pt/Pd mixture for 30 s to enhance contrast and prevent titania sample charging. Atomic force microscopy was conducted on a Veeco Dimension 5000 (Santa Barbara, CA) under tapping mode at a scan rate of 1 Hz.

### Nanodisc array fabrication

Titania nanofilms were constructed through layer-by-layer deposition of a titania precursor and polyelectrolyte, followed by calcination of the entire support.^9^ BK7 glass microscope slides were first cleaned using a boiling piranha solution (3 : 1 H_2_SO_4_ and 30% H_2_O_2_) for 30 min, followed by rinsing with nanopure water and drying under compressed air. The slides were then alternately soaked in PAH (1 mg ML^−1^, pH 7.5) and TALH (5 wt%, pH 7.5) for 1 min at a time, with 1 min rinses of nanopure water in between to build up a multilayer structure of electrostatically adsorbed compounds. After the desired number of layers was reached, the slide was thoroughly rinsed with nanopure water, dried under an N2 stream, and heated to 450 °C in a furnace for 4 h. After cooling to room temperature, the slide was rinsed alternately with nanopure water and ethanol three times.

Gold nanodisc (AuND) arrays were fabricated by hole-mask colloidal lithography (HCL) directly on the calcinated TiO2 films.^29^ A 4% PMMA solution in anisole was spin coated onto the substrate at 4000 rpm for 30 s, followed by soft baking at 170 °C for 10 min. The surface was plasma treated for 20 s in a Harrick PDC-32G source set to an RF power of 18 W, then immediately placed in a 0.2% PDDA solution for *ca.* 5 min before rinsing with nanopure water and drying under N_2_. Carboxylated polystyrene nanospheres (0.2% v/v) were applied to the PDDA-coated surface and incubated for 1 h, after which, the surface was rinsed with nanopure water and dried under N_2_. Thereafter, 20 nm of Au was deposited onto the surface using electron-beam evaporation (Temescal, Berkeley, CA) at 5 × 10^−6^ Torr in a Class 1000 cleanroom facility (UCR Center for Nanoscale Science & Engineering). The polystyrene spheres were removed by tape stripping (3M Scotch, USA), and the substrates were etched under O_2_ plasma (250 mTorr) in an STS MESC Multiplex RIE system (Newport, UK) set to an RF power of 50 W for 6 min. Finally, 2 nm of Cr and 50 nm of Au were deposited using electron-beam evaporation under the conditions described above, and the substrates were sonicated in acetone, leaving an evenly distributed array of AuNDs attached to the TiO2 surface. For array spot fabrication, patterning of distinct sensing areas was performed using a shadow mask during the final metal evaporation.^25^ Altogether, these methods exhibit excellent batch reproducibility, with the experiments herein representative of multiple, separate titania and AuND fabrications.

### MS sample preparation

An appropriate amount of β-casein stock solution (5 mg mL^−1^) in 50 mM NH_4_CO_3_ was digested with trypsin (1 mg mL^−1^ stock in 1 mM HCl) overnight at 37 °C. The final β-casein concentration was set to 1 mg mL^−1^ with a substrate : enzyme ratio of 50 : 1 (m/m).^30^ After completion of the digest, 40 μL of formic acid was added to the mixture to stop the enzymatic reaction. The sample was then diluted with nanopure water to the desired experimental concentration prior to spotting 1 μL droplets onto the titania/AuND surface. To provide even sample deposition, a hydrophobic corral of C18 was utilized for each array spot (ESI†).^9,21^ These sample droplets were incubated on-plate for 20 min within a humidity chamber at ambient temperature, followed by rinsing of the entire surface with 2% (w/w) TFA and drying under an N_2_ stream. Thereafter, 1 μL of a DHB matrix (10 mg mL^−1^) in 1 : 1 : 0.005 (v/v/v) ACN : H_2_O : H_3_PO_4_ was applied to each sample spot and dried in a vacuum desiccator. All substrates were fixed onto a custom stainless steel sample stage with conductive (5–10 mΩ) copper foil 1181 tape (3M, USA) for MS analysis.

## Results and discussion

### Design and fabrication

**Fig. 1:**
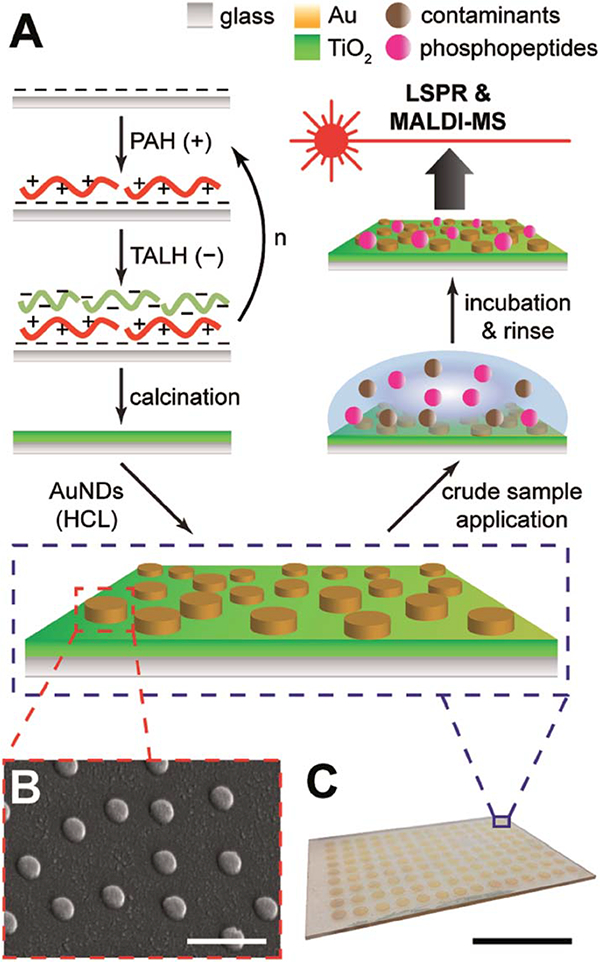
Fabrication and detection schemes for AuNDs on titania nanofilms. (A) Titania films are formed through layer-by-layer deposition of a polymer (PAH) and titania precursor (TALH), followed by calcination of the support and subsequent construction of AuNDs through hole-mask colloidal lithography (HCL). LSPR and MALDI-MS of phosphopeptides enriched from crude samples may then proceed on the substrate. (B) SEM of AuNDs on titania, scale bar represents 500 nm. (C) Representative array of nanodiscs, scale bar represents 25 mm.

Gold nanodiscs (AuNDs) on titania were fabricated by a layer-by-layer self-assembly/calcination process for the titania nanofilms, followed by hole-mask colloidal lithography of AuNDs on top of the substrate (Fig. 1A). The layer-by-layer (LbL) technique has been a long-established strategy for creating multilayer polyelectrolyte composites with tunable compositions and nanoscale thicknesses.^31^ If an inorganic precursor, such as silicate or Ti(IV), is incorporated into these assemblies and treated by calcination, any sacrificial organic polymers will combust, leaving a dense network of the inorganic oxide.^27^ This combination of LbL/calcination has been successfully applied in the fabrication of both titania and glass nanofilms ranging from 2–20 nm on both silica and gold supports.^9,20,27^ In the case of titania, a spray based method was used in the application of each layer, and with no rinsing of weakly adsorbed polyelectrolytes until the final layer had been applied, which resulted in a highly porous structure with microscale aggregates.^9^ While this provided an exceptionally large surface area for biomolecule enrichment, the high surface roughness prevents additional structures from being lithographically fabricated on top of the nanofilm since most commercial polymer resists may not evenly distribute. Therefore, the LbL/calcination method was modified herein for the formation of titania layers with a lower surface roughness that would be amenable to direct lithographic applications.

**Fig. 2:**
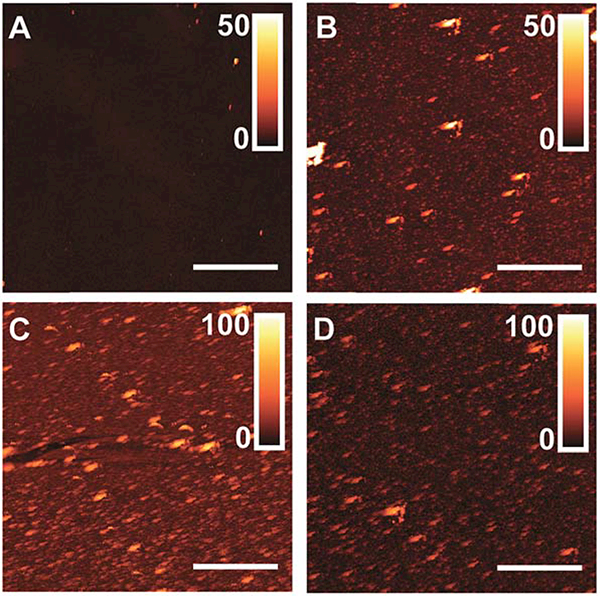
Atomic force microscopy images of titania nanofilms with varying (PAH/TALH)_*n*_ layers, post-calcination and rinsing. Color gradients (inset) represent z-axis height in nm, and scale bars represent 4 μm. (A) (PAH/TALH)_1_. (B) (PAH/TALH)_3_. (C) (PAH/TALH)_5_. (D) (PAH/ TALH)_10_.

Titania nanofilms of varying thickness and surface rough ness were constructed by alternate immersion of piranha-cleaned glass microscope slides in PAH and TALH, with copious rinsing of nanopure water in between each immersion step. This was tested for multiple deposition cycles (*n* = 1, 3, 5, and 10), and the resulting dried and calcinated substrates were investigated with atomic force microscopy (AFM, Fig. 2). As the (PAH/TALH)_*n*_ layer number increases, an increase in film thickness and surface roughness is observed up until (PAH/ TALH)_10_, in which a smoothing effect appears to take place (Table 1). There are also multiple aggregates of *ca.* 500 nm in length evident that increase in number with additional (PAH/ TALH)_*n*_ layers. The increase in surface roughness and aggregate density likely follows an Ostwald ripening mechanism due to PAH-induced bridging flocculation of TALH in (PAH/TALH)_1–5_, with Smoluchowski ripening (*i.e.* coalescing of aggregates) becoming dominant as the nanoscale clusters become more densely packed on the surface in (PAH/TALH)_10_.^32,33^ This is in stark contrast with titania fabricated through the spray LbL/ calcination method, which exhibit nano- and micropores (both absent here), massive aggregates of several microns in diameter, and surface roughnesses (root mean square, rms) of 23.7 nm for (PAH/TALH)_4_ and 116.8 nm for (PAH/TALH)_8_.^9^ In the immersion LbL/calcination method used here, the greatest surface roughness observed was 7.30 nm for (PAH/TALH)5 (Table 1), which has proven smooth enough for lithographic fabrication of gold nanostructures.

**Fig. 3:**
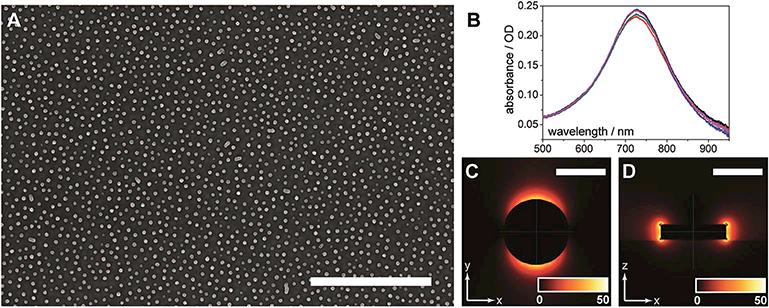
Characterization of gold nanodiscs. (A) SEM image of gold nanodiscs fabricated through hole-mask colloidal lithography (HCL), scale bar represents 2 μm. (B) LSPR spectra of AuNDs in air, exhibiting high reproducibility. Peak wavelength occurs at 727 ± 0.45 nm (*n* = 5 substrates). (C) Finite-difference time-domain (FDTD) simulation of electromagnetic (EM) field distribution (top view), color gradient (inset) represents EM intensity in arbitrary units, and scale bar represents 150 nm. (D) FDTD simulation of EM field distribution (side view), color gradient (inset) represents EM intensity in arbitrary units, and scale bar represents 150 nm.

The AuNDs were fabricated through hole-mask colloidal lithography, a versatile technique utilizing uniformly adsorbed polystyrene nanospheres on a PMMA resist to create a self- assembled mask for metal deposition in distinct nanoscale areas.^29^ Here, a PMMA resist was applied directly to the calcinated titania film and HCL was performed on top of the fabricated substrate (Fig. 1). The resulting AuNDs were thereafter subjected to morphological and optical characterizations (Fig. 3). Scanning electron microscopy reveals the AuNDs to be remarkably monodisperse and evenly distributed across the entire nanofilm, with no evident voids of AuNDs, and minimal aggregation into dimer or trimer clusters (Fig. 1B and 3A). This is reflected in their localized surface plasmon resonance (LSPR) absorbance spectra (Fig. 3B), which display only a 0.06% relative standard deviation in their peak position across an entire 50 × 75 mm glass/titania support (λ_peak_ = 727 ± 0.45 nm, *n*= 5). The localized surface plasmons of the nanodiscs were modeled using finite-difference time-domain simulations, which reveal the generated electromagnetic fields to be concentrated at the tips of the structures, extending *ca.* 50 nm away from the surface, with the highest intensities within 20 nm of the surface (Fig. 3C and D). This is in general agreement with standard LSPR mechanisms,^16^ and allows for label-free spectroscopic sensing to take place in close vicinity of the nanodiscs, in addition to enhanced laser desorption/ionization in surface mass spectrometric measurements.^20,21^

### Enrichment capacities of multilayer composites

**Fig. 4:**
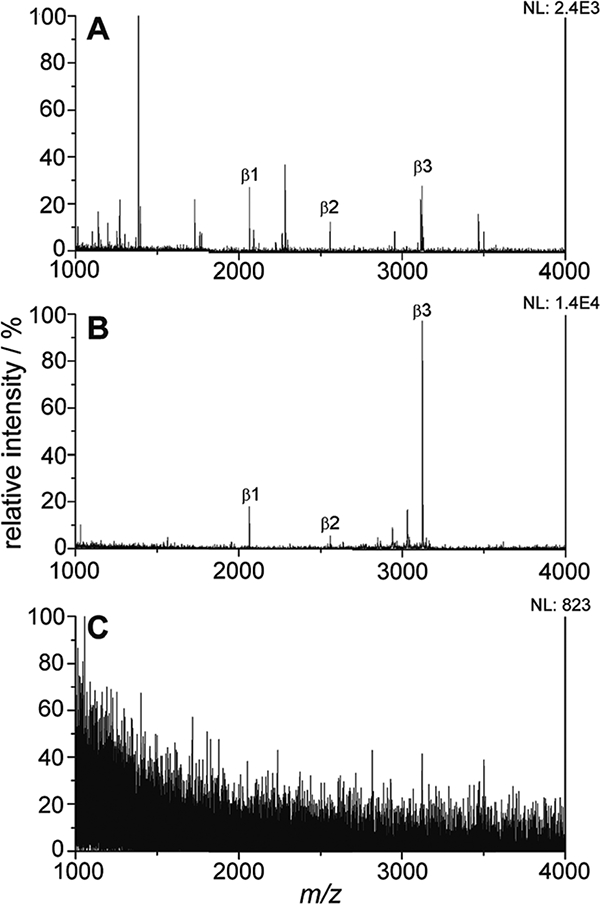
On-plate enrichment of phosphorylated peptides from a β- casein digest. (A) MALDI mass spectrum of ß-casein peptides produced by overnight digestion with trypsin. (B) Phosphorylated species enriched on the titania/AuND surface for 20 min, with contaminants removed through rinsing. (C) Incubation of the β-casein digest on a standard glass/AuND surface results in no retention of phosphopeptides after rinsing.

The titania/AuND composite was tested for its ability to enrich phosphorylated species from a complex peptide/protein mixture. For these initial studies, the (PAH/TALH)_5_ substrate was utilized, and variations in layer number were investigated thereafter. The mass spectra of a tryptic digest of β-casein in the amount of 10 pmol on a standard stainless steel MALDI plate, the titania/AuND composite, and an unmodified glass slide are provided in Fig. 4. β-Casein is known to contain multiple phosphorylation sites, particularly at serine residues, though targeted studies investigating the location and extent of these phosphorylations remain challenging when other species competing for ionization are present. This is evident within the mass spectrum of the digest on a standard MALDI plate, which exhibits multiple peaks attributed to non-phosphorylated peptides and digest contaminants, in addition to three main phosphorylated species (Fig. 4A). These peaks, at *m/z* 2062, 2557, and 3122, have been denoted as β1, β2, and β3, respectively, and their sequences and relative positions are provided in Table 2. All three phosphopeptides are of relatively low signal abundance (<40%) compared to the other species present, rendering selective characterization difficult. Enrichment of the b-casein digest on the titania/AuND composite, on the other hand, yields a far more simplified spectrum (Fig. 4B). After a 20 min incubation on this substrate, with rinsing of non-specifically adsorbed contaminants thereafter, the β1, β2, and b3 peaks become far more prominent over the background, with the b3 peak dominant above the other two. This is attributed to β3 having four phosphorylated serine residues, as opposed to b1 and b2 having one phosphorylation site each (Table 2), thus resulting in multivalent binding of β3 to the titania/AuND surface. The presence of these additional phosphorylation sites is further apparent in the analysis of the fragment peaks between *m/z* 2830–3027, denoted as β3a, β3b, and β3c. Indistinguishable in the standard MALDI data, each of these peaks occurs *ca. m/z* 97 lower than one another, suggesting successive losses of phosphate from the b3 parent ion. This series of fragmentations is likely caused by the ionization laser source, and if desired, may be mitigated through the use of lower laser fluences.^34^ The enrichment process on the titania/ AuND surface is quite important in the assignment of the parent ion to the β3a-c peaks, which could be precluded in the presence of interferents and without MS/MS or high-resolution instrumentation. To verify that the enrichment can be attributed to the titania nanofilm and not the glass support or gold nanostructures, an AuND on glass (no titania) substrate was subjected to the same incubation and enrichment process for β- casein, though no MS peaks could be detected after the rinsing and analysis steps (Fig. 4C).

When the effect of (PAH/TALH)_*n*_ layer number on the enrichment capacity of the titania/AuND substrates was investigated, a general correlation between phosphopeptide affinity and apparent surface coverage of titania was observed (Table 1 and Fig. S1†). Increasing the (PAH/TALH)_n_ layer number from *n* = 1–10 resulted in gradual increases in the ionization intensities of the ß1, ß2, and ß3 MS peaks, with the signals plateauing around 5 (PAH/ TALH)_*n*_ layers (Fig. S1†). While this data suggests that full surface coverage is not achieved until at least 5 layers of polyelectrolytes have been deposited, further analyses were required to characterize the distribution of titania on the final calcinated surfaces.

**Fig. 5:**
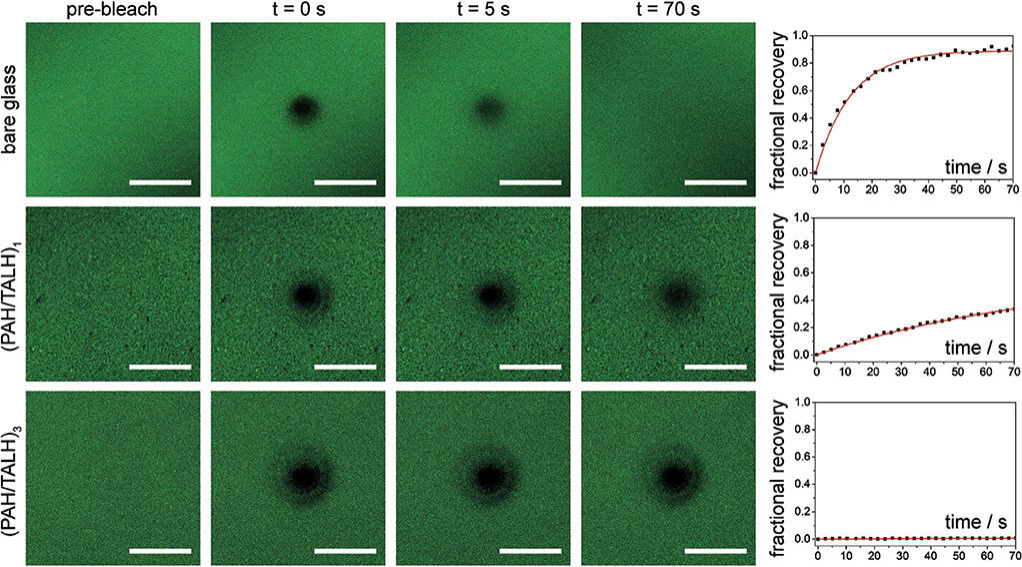
Lateral mobility studies of supported phospholipid membranes. Fluorescence recovery after photobleaching (FRAP) microscopy images before, during, and after fluorophore tagged lipid bleaching on bare glass, (PAH/TALH)_1_, and (PAH/TALH)_3_, with associated recovery curves.

Common nanoscale visualization and identification techniques, such as SEM coupled with energy dispersive X-ray spectroscopy, can be problematic for investigating homeogenous distributions of titania due to the tendency of the material to charge under the electron beam without a conductive coating.^35^ To circumvent this issue, an indirect visualization method involving fluorescently tagged supported phospholipid membranes was utilized. When a suspension of small, unilamellar lipid vesicles (SUVs) is applied to a hydrophilic support, vesicles are known to electrostatically adsorb, and in the case of glass, rupture, fuse, and self-assemble into a supported lipid bilayer.^36^ Fluorescence recovery after photobleaching (FRAP) has traditionally been used to investigate the mobility of fluorophore-tagged lipids embedded within supported membranes, with those on glass exhibiting high lateral mobility.^37^ Titania, on the other hand, has been shown to only adsorb intact vesicles, with their rupture only induced by viral peptides or pH adjustment.^38–40^ Without these rupture techniques, titania-supported vesicles will exhibit no long-range lateral mobility of embedded phospholipids. With this in mind, fluorescent phosphocholine SUVs were applied to bare glass and the (PAH/TALH)_1–10_ substrates, and the surfaces were thereafter subjected to analysis by FRAP (see ESI† for experimental details). Confocal microscopy reveals an even distribution of fluorescence across all surfaces prior to photo-bleaching, indicative of uniform adsorption of SUVs to glass and calcinated titania (Fig. 5 and S2†). After photobleaching, recovery of fluorescence within the bleached regions, indicative of lateral phospholipid mobility, is noted on the glass and (PAH/TALH)_1_ substrates, albeit with a decreased mobile fraction on (PAH/ TALH)_1_, with the (PAH/TALH)_3–10_ substrates exhibiting no redistribution of fluorescence (Table 1, Fig. 5 and S2†).

Taken together with the AFM (Fig. 2) and MS (Fig. S1†) results, it can be inferred that full surface coverage of titania is obtained at n = 3 calcinated (PAH/TALH)_*n*_ layers, with (PAH/ TALH)1 exhibiting voids of exposed glass that allow for vesicle rupture and lateral lipid mobility. These voids in titania limit the phosphopeptide enrichment capacity of the final substrate, which plateaus at *n* = 5 (PAH/TALH)_*n*_ layers. The increase in enrichment capacity and ß1-ß3 ionization intensity between (PAH/TALH)3 and (PAH/TALH)5 could be due to the increase of surface roughness in (PAH/TALH)5, granting a greater surface area for biomolecule adsorption (Table 1). This layer number was therefore chosen as optimal for all subsequent phospho-peptide analyses.

### *In situ* multimodal monitoring and detection

**Fig. 6:**
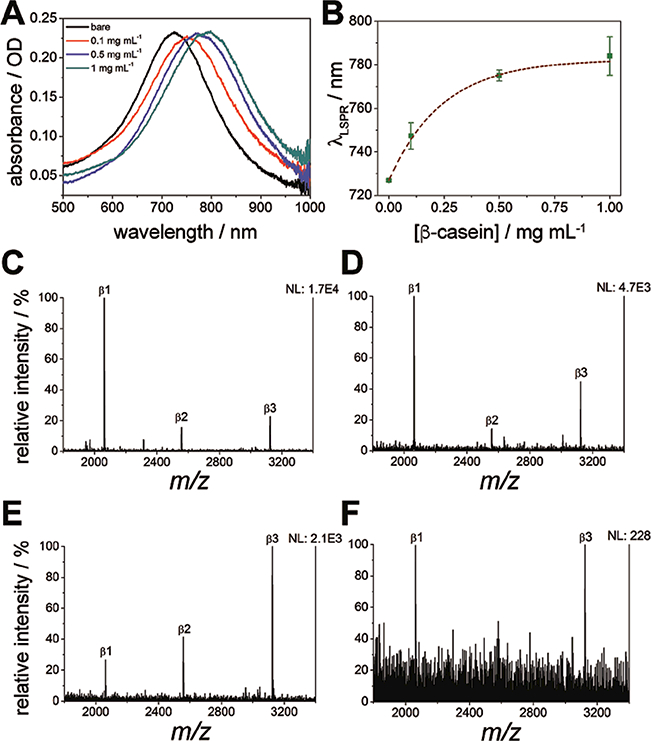
Phosphopeptide enrichment monitored with LSPR and MALDI-MS. (A) Representative LSPR spectra of varying concentrations of protein digest deposited on the titania/AuND surface. (B) LSPR wavelength shifts from varying concentrations of protein digest deposited on the titania/ AuND surface. Error bars are the result of *n* = 5 measurements. (C-F) Enriched β-casein digests in the amount of: 10 pmol (C), 1 pmol (D), 900 fmol (E), and 800 fmol (F).

Localized surface plasmon resonance (LSPR) spectroscopy of the nanodiscs on calcinated titania was used to monitor the enrichment of phosphopeptides on the substrate, in addition to the removal of non-specifically adsorbed salts and biomolecules. Absorbance spectra of the titania/AuND arrays under varying ß-casein digest loadings (0–1 mg mL^−1^) are provided in Fig. 6A. Noteworthy in this data (Fig. 6A and B) is that each starting peak wavelength (0 mg mL^−1^, *ca. 727* nm) remained constant, and is in close agreement with the nanodisc array spectra from other LSPR experiments (Fig. 3B), despite the AuNDs and titania being fabricated in separate batches and on different days. Each increase in digest concentration results in a shift in the nanodisc absorbance peak toward higher wavelengths, and the amount of bulk sample can be relatively quantified from these shifts with minimal deviation in response (Fig. 6B). It is noted that above 0.25 mg mL^−1^ of β-casein, the range of linearity for the curve is exceeded and the LSPR signal begins to saturate, which may be due to the low penetration depth (<50 nm, Fig. 3C and D) of localized surface plasmons generated by the nanodiscs. For practical considerations, this data also indicates that accurate quantitation of bulk peptide/ protein solutions should only take place below this level. Following rinsing of these surfaces with 2% TFA and drying under N_2_, all absorbance spectra return to their original peak wavelength (*727* nm), suggesting that nearly all components of the mixture have been removed from the substrate. While it can be inferred from the LSPR results alone that no biomolecules remain, MALDI-MS was thereafter applied for confirmation and identification of any remaining species (Fig. 6C-F). Indeed, the phosphorylated species from β-casein, ß1-ß3, can be successfully detected on these substrates down to a level of 900 fmol of applied digest (Fig. 6E). Below this level (*i.e.* 800 fmol, Fig. 6F) only the β1 and β3 peaks can be identified above an acceptable signal-to-noise level (S/N > 3). Taking the above results together, the titania substrate fabricated here through LbL/calcination, and in a manner that renders it capable of supporting lithographically patterned nanoparticles, exhibits promise as a chip-based platform for multimodal phosphoproteome characterization. An integrated MS substrate with online and label-free detection capabilities offered by LSPR can be envisioned, with many opportunities open for exotic nanofabrication techniques and sensitivity enhancements.

## Conclusions

The chosen application method for the construction of LbL/ calcinated substrates has previously been shown to have a dramatic impact on the morphology of the final nanofilms.^27,28^ Herein we have investigated this issue for assemblies constructed from TALH and PAH, resulting in titania nanofilms optimized for direct lithographic nanoparticle fabrications. In using an immersion based application of polyelectrolytes, as opposed to a spray based method, surface roughness of the calcinated products was substantially minimized, yet still retained the ability to enrich phosphorylated species with appreciable sensitivity under MALDI-MS analysis. Importantly, application of gold nanodiscs to these titania surfaces by hole-mask colloidal lithography proved successful, with all titania/ AuND arrays exhibiting excellent uniformity in nanoparticle morphology and optical properties. As a demonstration of how hyphenated LSPR and MALDI-MS can be carried out, LSPR absorbance shifts from the nanodiscs were used to monitor the bulk application and removal of a tryptic digest of ß-casein, with MALDI-MS confirming the capture of lowly abundant phosphopeptides within the sample. As future platforms are designed for clinical and field analyses, higher sensitivities in LSPR measurements will surely be desired so that optical quantitation of the enriched species can also take place. One method to accomplish this may be through the use of alternative nanoparticle geometries that alter the plasmonic field and resulting LSPR sensitivity, which may include cones, rings, crescents, and nonconcentric features deposited using clean-room fabrication techniques,^29,41–44^ in addition to lithographically patterned rings deposited *via* newly developed electrochemical deposition protocols.^45,46^ Additionally, within the present study nanodiscs remained bare with no protective monolayer or coating, thus resulting in all phosphopeptides being captured on the titania between the gold structures. While beneficial for monitoring the removal of bulk contaminants, application of thin (<20 nm) titania films directly to the nanodisc surface could be advantageous for enhanced capture of phosphorylated species within the penetration depth of the localized surface plasmons generated. Once the LSPR aspects are refined and further developed, future testing should commence using cultured cell systems, with data gathering proceeding for established phosphorylation pathways, eventually paving the way for biological discoveries. With the above in mind, the calcinated titania nanofilms fabricated here grant exceptional versatility as an underlayment for lithographic nanofabrication techniques, which will certainly prove effective for hyphenated, chip-based, and label-free analyses in phos-phoproteome research, given that patterned LSPR arrays are capable of highly reproducible measurements, with complementary identification capabilities offered by surface based mass spectrometry.

## Supplementary Material

ESI

## Figures and Tables

**Table 1:** Physical Properties of Multilayer Titania Nanofilms

(PAH/TALH)_*n*_ layers	0	1	3	5	10
Surface roughness (nm)^a^	—	1.39	4.80	7.30	5.09
Phosphopeptide enrichment	None	Weak	Weak	Strong	Strong
Phospholipid mobility^b^	100% (3.0 μm^2^ s^−1^)	63% (1.9 μm^2^ s^−1^)	0%	0%	0%

a Surface roughness is provided as the root mean square (rms) of the height profile over a 10 μm^2^ area, measured by AFM.

b Mobilities of supported lipids are provided as the mobile fraction (β) in percent of total lipids bound to the substrate. If lipids were found to be mobile, then their diffusion coefficients (*D*) were also provided.

**Table 2:** Identified phosphopeptides from tryptic digestion of β-casein

Peptide name	[M + H]^+^ *m/z* (theoretical)^a^	[M + H]^+^ *m/z* (experimental)	Amino acid sequence^b^	Position
β1	2061.83	2062	FQ[pS]EEQQQTEDELQDK	48–63
β2	2556.09	2557	FQ[pS]EEQQQTEDELQDKIHPF	48–67
β3	3122.26	3122	RELEELNVPGEIVE[pS]L[pS][pS][pS]EESITR	16–40

a Theoretical masses determined with the PeptideMass tool in ExPASy (Expert Protein Analysis System, http://www.uniprot.org) using UniProtKB data for β-casein (entry ID: P02666).

b [pS] represents a phosphorylated serine residue, and is included in the theoretical mass calculation.

## Footnotes

† Electronic supplementary information (ESI) available: Additional methods and figures. See DOI: 10.1039/c7ra08870a

## References

[1] Olsen JV, Blagoev B, Gnad F, Macek B, Kumar C, Mortensen P and Mann M, Cell, 2006, 127, 635–648. 17081983 10.1016/j.cell.2006.09.026

[2] Riley NM and Coon JJ, Anal. Chem, 2016, 88, 74–94. 26539879 10.1021/acs.analchem.5b04123 PMC4790442

[3] Wang ZG, Lv N, Bi WZ, Zhang JL and Ni JZ, ACS Appl. Mater. Interfaces, 2015, 7, 8377–8392. 25845677 10.1021/acsami.5b01254

[4] Connor PA and McQuillan AJ, Langmuir, 1999, 15, 2916–2921.

[5] Leitner A, TrAC, Trends Anal. Chem, 2010, 29, 177–185.

[6] Nawrocki J, Dunlap C, McCormick A and Carr PW, J. Chromatogr. A, 2004, 1028, 1–30. 14969280 10.1016/j.chroma.2003.11.052

[7] Nawrocki J, Dunlap C, Li J, Zhao J, McNeff CV, McCormick A and Carr PW, J. Chromatogr. A, 2004, 1028, 31–62. 14969281 10.1016/j.chroma.2003.11.050

[8] Qiao L, Roussel C, Wan JJ, Yang PY, Girault HH and Liu AH, J. Proteome Res, 2007, 6, 4763–4769. 18047269 10.1021/pr0705284

[9] Wang H, Duan JC and Cheng Q, Anal. Chem, 2011, 83, 1624–1631. 21306131 10.1021/ac1024232 PMC7360113

[10] Chen CJ, Lai CC, Tseng MC, Liu YC, Liu YH, Chiou LW and Tsai FJ, Anal. Chim. Acta, 2014, 812, 105–113. 24491770 10.1016/j.aca.2014.01.010

[11] Hsieh HC, Sheu C, Shi FK and Li DT, J. Chromatogr. A, 2007, 1165, 128–135. 17714720 10.1016/j.chroma.2007.08.012

[12] Tan YJ, Sui DX, Wang WH, Kuo MH, Reid GE and Bruening ML, Anal. Chem, 2013, 85, 5699–5706. 23638980 10.1021/ac400198n PMC3721342

[13] Wang H, Duan YK and Zhong WW, ACS Appl. Mater. Interfaces, 2015, 7, 26414–26420. 26571083 10.1021/acsami.5b09348

[14] Najam-ul-Haq M, Jabeen F, Fatima B, Ashiq MN and Hussain D, Amino Acids, 2016, 48, 2571–2579. 27339789 10.1007/s00726-016-2281-5

[15] Sturm M, Leitner A, Smatt JH, Linden M and Lindner W, Adv. Funct. Mater, 2008, 18, 2381–2389.

[16] Willets KA and Van Duyne RP, Annu. Rev. Phys. Chem, 2007, 58, 267–297. 17067281 10.1146/annurev.physchem.58.032806.104607

[17] Anker JN, Hall WP, Lyandres O, Shah NC, Zhao J and Van Duyne RP, Nat. Mater, 2008, 7, 442–453. 18497851 10.1038/nmat2162

[18] Unser S, Bruzas I, He J and Sagle L, Sensors, 2015, 15, 15684–15716. 26147727 10.3390/s150715684 PMC4541850

[19] Anker JN, Hall WP, Lambert MP, Velasco PT, Mrksich M, Klein WL and Van Duyne RP, J. Phys. Chem. C, 2009, 113, 5891–5894. 10.1021/jp900266k PMC2723955 20161175

[20] Chen C-Y, Hinman SS, Duan J and Cheng Q, Anal. Chem, 2014, 86, 11942–11945. 25417963 10.1021/ac503808r PMC4270398

[21] Hinman SS, Chen CY, Duan J and Cheng Q, Nanoscale, 2016, 8, 1665–1675. 26694584 10.1039/c5nr06635b PMC5412507

[22] Lin HY, Chen CT and Chen YC, Anal. Chem, 2006, 78, 6873–6878. 17007509 10.1021/ac060833t

[23] Chen JY and Chen YC, Anal. Bioanal. Chem, 2011, 399, 1173–1180. 21058028 10.1007/s00216-010-4397-x

[24] Sepulveda B, Angelome PC, Lechuga LM and Liz- Marzan LM, Nano Today, 2009, 4, 244–251.

[25] Ruemmele JA, Hall WP, Ruvuna LK and Van Duyne RP, Anal. Chem, 2013, 85, 4560–4566. 23560643 10.1021/ac400192f PMC3696404

[26] He J, Boegli M, Bruzas I, Lum W and Sagle L, Anal. Chem, 2015, 87, 11407–11414. 26494412 10.1021/acs.analchem.5b02870

[27] Phillips KS, Han JH, Martinez M, Wang ZZ, Carter D and Cheng Q, Anal. Chem , 2006, 78, 596–603. 16408945 10.1021/ac051644y

[28] Linman MJ, Culver SP and Cheng Q, Langmuir, 2009, 25, 3075–3082. 19437774 10.1021/la803835a

[29] Fredriksson H, Alaverdyan Y, Dmitriev A, Langhammer C, Sutherland DS, Zaech M and Kasemo B, Adv. Mater, 2007, 19, 4297–4302.

[30] Wang H, Duan JC, Zhang LH, Liang Z, Zhang WB and Zhang YK, J. Sep. Sci, 2008, 31, 480–487. 18210378 10.1002/jssc.200700445

[31] Decher G, Science, 1997, 277, 1232–1237.

[32] Krost A, Christen J, Oleynik N, Dadgar A, Deiter S, Blasing J, Krtschil A, Forster D, Bertram F and Diez A, Appl. Phys. Lett, 2004, 85, 1496–1498.

[33] Thiel PA, Shen M, Liu DJ and Evans JW, J. Phys. Chem. C, 2009, 113, 5047–5067. 10.1063/1.3078033 19275412

[34] Wu QH, Pomerantz AE, Mullins OC and Zare RN, J. Am. Soc. Mass Spectrom, 2013, 24, 1116–1122. 23633019 10.1007/s13361-013-0636-7

[35] Kim KH, Akase Z, Suzuki T and Shindo D, Mater. Trans, 2010, 51, 1080–1083.

[36] Castellana ET and Cremer PS, Surf. Sci. Rep, 2006, 61, 429–444. 10.1016/j.surfrep.2006.06.001 PMC7114318 32287559

[37] Axelrod D, Koppel DE, Schlessinger J, Elson E and Webb WW, Biophys. J, 1976, 16, 1055–1069. 786399 10.1016/S0006-3495(76)85755-4 PMC1334945

[38] Cho NJ, Cho SJ, Cheong KH, Glenn JS and Frank CW, J. Am. Chem. Soc, 2007, 129, 10050–10051. 17661464 10.1021/ja0701412

[39] Cho NJ and Frank CW, Langmuir, 2010, 26, 15706–15710. 20857902 10.1021/la101523f

[40] Zan GH, Jackman JA, Kim SO and Cho NJ, Small, 2014, 10, 4828–4832. 25079046 10.1002/smll.201400518

[41] Cataldo S, Zhao J, Neubrech F, Frank B, Zhang C, Braun PV and Giessen H, ACS Nano, 2012, 6, 979–985. 22176349 10.1021/nn2047982

[42] Horrer A, Schafer C, Broch K, Gollmer DA, Rogalski J, Fulmes J, Zhang D, Meixner AJ, Schreiber F, Kern DP and Fleischer M, Small, 2013, 9, 3987–3992. 24302595 10.1002/smll.201300449

[43] Syrenova S, Wadell C and Langhammer C, Nano Lett, 2014, 14, 2655–2663. 24697350 10.1021/nl500514y

[44] Zhao Z, Cao Y, Cai Y, Yang J, He X, Nordlander P and Cremer PS, ACS Nano, 2017, 11, 6594–6604. 28704035 10.1021/acsnano.6b07867

[45] Halpern AR and Corn RM, ACS Nano, 2013, 7,1755–1762. 23330883 10.1021/nn3058505

[46] Cho K, Loget G and Corn RM, J. Phys. Chem. C, 2014, 118, 28993–29000. 10.1021/jp501783z PMC4275153 25553204

