# Supplementary material for: Plasmonic nanodisc arrays on calcinated titania for multimodal analysis of phosphorylated peptides
Source: RSC Adv. Author manuscript; Available in PMC 2019 Jan 28. (PMC6349370; doi:10.1039/C7RA08870A)
Supplement: ESI [file NIHMS978994-supplement-ESI.pdf]

Electronic Supplementary Material (ESI) for RSC Advances.
This journal is © The Royal Society of Chemistry 2017

# †ELECTRONIC SUPPLEMENTARY INFORMATION (ESI)

# *Plasmonic Nanodisc Arrays on Calcinated Titania for Multimodal Analysis of Phosphorylated Species*

Samuel S. Hinman,[a] Romie C. T. Nguyen,[b] and Quan Cheng[ab]*

[a]Environmental Toxicology and [b]Department of Chemistry, University of California – Riverside, Riverside, CA 92521, USA

*E-mail: quan.cheng@ucr.edu

**Additional Experimental Methods** . . . . . . . S-2

Patterning of Hydrophobic C18 Corrals . . . . . . S-2

Lipid Vesicle Preparation . . . . . . . . S-2

Fluorescence Microscopy . . . . . . . . S-2

FRAP Analysis . . . . . . . . . S-3

**Supplementary Figures** . . . . . . . . S-4

Figure S1. Phosphopeptide Enrichment Mass Spectra . . . . S-4

Figure S2. FRAP of Supported Lipids on Titania . . . . . S-5

**References** . . . . . . . . . . S-6

## ■ SUPPLEMENTARY METHODS

**Patterning of Hydrophobic C18 Corrals**

Titania was modified with C18 and photopatterned using a modification of established methods.[1, 2] The titania coated microscope slides were rinsed alternately with hexanes and ethanol three times. Thereafter, the slides were immersed in 10 mM OTS in hexanes to generate a hydrophobic C18 layer, then rinsed alternately with hexanes and ethanol three more times before drying under a stream of nitrogen gas. The entire glass/titania/C18 substrate was covered by a shadow mask and irradiated under a 450 mW cm$^{-2}$, $\lambda$ = 254 nm, light source (CL-1000, UVP Inc., Upland, CA) for one hour to cleave C18 from the exposed areas, and obtain a pattern of hydrophilic spots with hydrophobic surroundings. The shadow mask was perforated with holes representing the final array, separated into areas of 2 mm dia. spots with 3 mm pitch, 1 mm dia. spots with 3 mm pitch, and 800 µm dia. spots with 1.5 mm pitch.

**Lipid Vesicle Preparation**

An appropriate amount of POPC and 2% (w/w) NBD-PC stock solution in chloroform was dried in a glass vial under nitrogen to form a thin lipid film. The vial containing lipids was then placed in a vacuum desiccator for at least 2 h to remove any residual solvent. The dried lipids were resuspended in 1×PBS to a lipid concentration of 1.0 mg mL$^{-1}$. After vigorous vortexing to remove all lipid remnants from the vial wall, the solution was bath sonicated for 30 min. Thereafter, the supernatant was extruded through a polycarbonate filter (Whatman, 100 nm) to produce small, unilamellar vesicles (SUVs) of uniform size. All vesicle suspensions were used within one week and stored at 4 °C.

**Fluorescence Microscopy**

Fluidity of membranes incorporating the fluorescent phospholipids was examined using fluorescence recovery after photobleaching (FRAP). Supported lipid bilayers were formed on clean glass coverslips (Fisher Scientific, Pittsburgh, PA) using vesicle suspensions deposited in 4.5 mm PDMS wells on the glass surface. These were incubated for 1 h prior rinsing with copious amounts of nanopure water. To assist with identification of the bilayer focal plane, a peripheral scratch in the bilayer was made. Fluorescence microscopy was carried out on an inverted Leica TCS SP5 II (Leica Microsystems, Buffalo Point, IL) using the 488 nm Argon laser line in conjunction with a 40× (NA 1.1) objective and Leica HyD hybrid detector.

Photobleaching at 1.5 mW for 500 ms and fluorescence recovery monitoring were set up and performed using the LAS AF software package as described previously.[3-5]

**FRAP Analysis**

The methods of Axelrod and Soumpasis were applied to derive diffusion coefficients for each membrane.[3, 4] First, the fluorescence intensity of each bleach spot was normalized over a background area of the same size to account for background photobleaching. This normalized value ($F_n$) was then used within the following formula to obtain the FRAP ratio ($F_{FRAP}$), with $F_0$ being the normalized intensity of the bleached area immediately after bleaching.

$$F_{FRAP} = \frac{F_n - F_0}{1 - F_0}$$

Thereafter, $F_{FRAP}$ was plotted against time and fitted to a first order exponential function. The diffusion coefficient was calculated using the following equation, with $D$ being the diffusion coefficient, $\omega$ the full width at half maximum of the focused Gaussian laser profile, $t_{1/2}$ the half-time recovery obtained from the exponential fit, and $\gamma$ a correction factor accounting for the laser beam geometry.

$$D = \frac{\omega^2}{4t_{1/2}}\gamma$$

## ■ SUPPLEMENTARY FIGURES

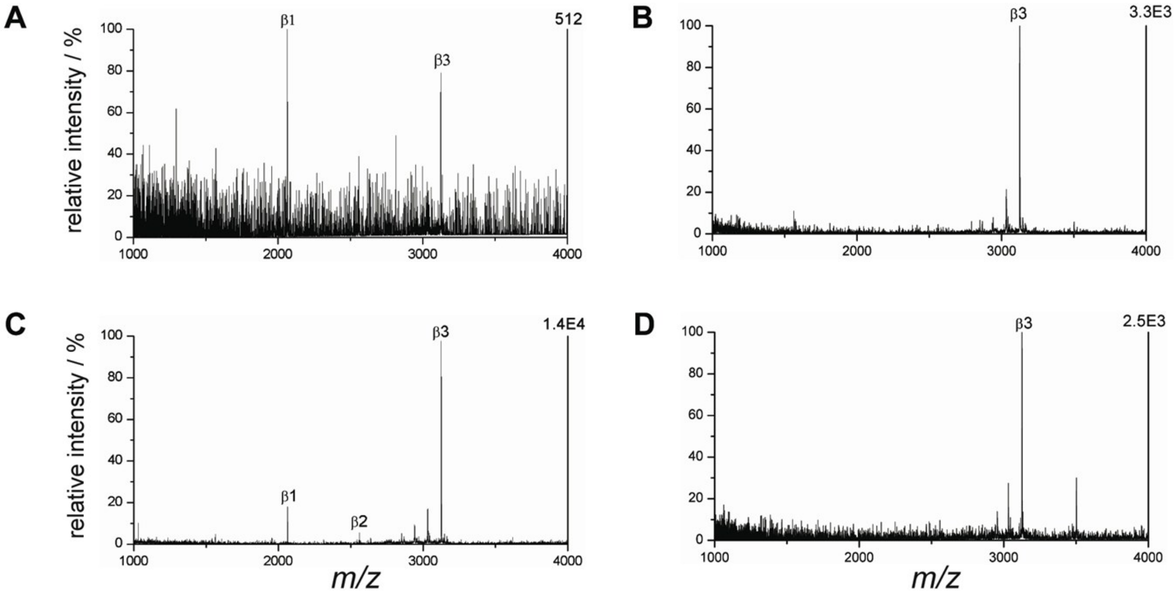


**Fig. S1** On-plate enrichment of phosphorylated peptides from a β-casein digest on varying $(PAH/TALH)_n$ layers. (A) $(PAH/TALH)_1$. (B) $(PAH/TALH)_3$. (C) $(PAH/TALH)_5$. (D) $(PAH/TALH)_{10}$

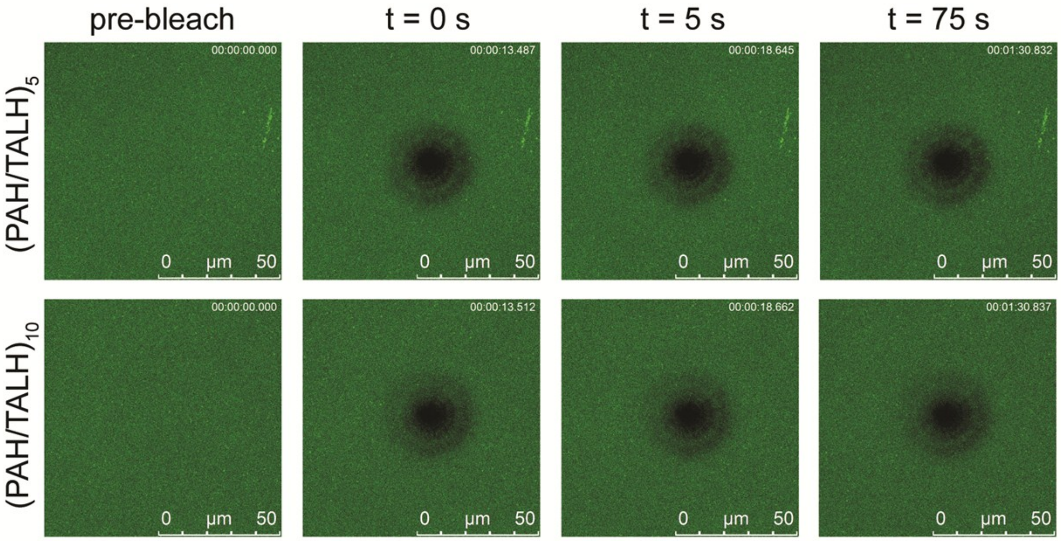


**Fig. S2** FRAP analysis of supported lipid membranes on additional (PAH/TALH)$_n$ layers.

## ■ REFERENCES


1 H. Wang, J. C. Duan and Q. Cheng, *Anal. Chem.*, 2011, **83**, 1624-1631.

2 S. S. Hinman, C. Y. Chen, J. Duan and Q. Cheng, *Nanoscale*, 2016, **8**, 1665-1675.

3 D. Axelrod, D. E. Koppel, J. Schlessinger, E. Elson and W. W. Webb, *Biophys. J.*, 1976, **16**, 1055-1069.

4 D. M. Soumpasis, *Biophys. J.*, 1983, **41**, 95-97.

5 S. S. Hinman, C. J. Ruiz, G. Drakakaki, T. E. Wilkop and Q. Cheng, *ACS Appl. Mater. Interfaces*, 2015, **7**, 17122-17130.
